# Supplementary material for: De novo macrocyclic peptides dissect energy coupling of a heterodimeric ABC transporter by multimode allosteric inhibition
Source: eLife. 2021 Apr 30;10:e67732. doi: 10.7554/eLife.67732 (PMC8116058; doi:10.7554/eLife.67732)

| Figure 2 | a |  |  |  |
| --- | --- | --- | --- | --- |
|  |  |  |  |  |
| TmrAB in liposomes | |  | Bound CP6F |  |
| µM |  |  | % |  |
|  |  |  | Mean | SD |
|  |  |  |  |  |
| 0.0000 |  |  | 0.00 | 2.66 |
| 0.0025 |  |  | 23.95 | 1.95 |
| 0.0250 |  |  | 50.23 | 2.48 |
| 0.0500 |  |  | 67.16 | 1.90 |
| 0.1250 |  |  | 86.81 | 1.95 |
| 0.2500 |  |  | 91.77 | 3.69 |
| 0.3750 |  |  | 95.92 | 2.63 |
| 0.5000 |  |  | 94.60 | 2.74 |
|  |  |  |  |  |
| Empty liposomes | |  | Bound CP6F |  |
| µM |  |  | % |  |
|  |  |  | Mean | SD |
|  |  |  |  |  |
| 0.5000 |  |  | -6.19 | 9.90 |
|  |  |  |  |  |
|  |  |  |  |  |
| TmrAB in liposomes | |  | Bound CP12F | |
| µM |  |  | % |  |
|  |  |  | Mean | SD |
| 0.0000 |  |  | 0.00 | 0.48 |
| 0.0025 |  |  | 21.56 | 0.75 |
| 0.0250 |  |  | 55.19 | 1.11 |
| 0.0500 |  |  | 68.59 | 2.85 |
| 0.1250 |  |  | 78.78 | 1.92 |
| 0.2500 |  |  | 87.50 | 2.60 |
| 0.3750 |  |  | 94.67 | 0.98 |
| 0.5000 |  |  | 100.00 | 1.49 |
|  |  |  |  |  |
| Empty liposomes | |  | Bound CP12F | |
| µM |  |  | % |  |
|  |  |  | Mean | SD |
|  |  |  |  |  |
| 0.5000 |  |  | -0.19 | 0.41 |

| Figure 2 | b |  |  |  |
| --- | --- | --- | --- | --- |
|  |  |  |  |  |
| TmrAB in liposomes | |  | Bound CP13F | |
| µM |  |  | % |  |
|  |  |  | Mean | SD |
|  |  |  |  |  |
| 0.0000 |  |  | 0.00 | 2.96 |
| 0.0025 |  |  | 13.77 | 2.41 |
| 0.0500 |  |  | 46.57 | 2.38 |
| 0.1250 |  |  | 72.25 | 5.37 |
| 0.2500 |  |  | 85.91 | 2.78 |
| 0.5000 |  |  | 88.99 | 4.37 |
|  |  |  |  |  |
| Empty liposomes | |  | Bound CP13F | |
| µM |  |  | % |  |
|  |  |  | Mean | SD |
|  |  |  |  |  |
| 0.2500 |  |  | 3.65 | 2.10 |
|  |  |  |  |  |
|  |  |  |  |  |
| TmrAB in liposomes | |  | Bound CP14F | |
| µM |  |  | % |  |
|  |  |  | Mean | SD |
|  |  |  |  |  |
| 0.0000 |  |  | 0.00 | 3.33 |
| 0.0005 |  |  | 13.49 | 2.37 |
| 0.0050 |  |  | 26.27 | 3.32 |
| 0.0250 |  |  | 55.22 | 2.38 |
| 0.0500 |  |  | 77.37 | 4.94 |
| 0.1000 |  |  | 85.31 | 5.50 |
| 0.2000 |  |  | 93.45 | 3.18 |
|  |  |  |  |  |
| Empty liposomes | |  | Bound CP14F | |
| µM |  |  | % |  |
|  |  |  | Mean | SD |
|  |  |  |  |  |
| 0.2500 |  |  | -5.24 | 2.64 |

| Figure 2 | c |  |  |  |
| --- | --- | --- | --- | --- |


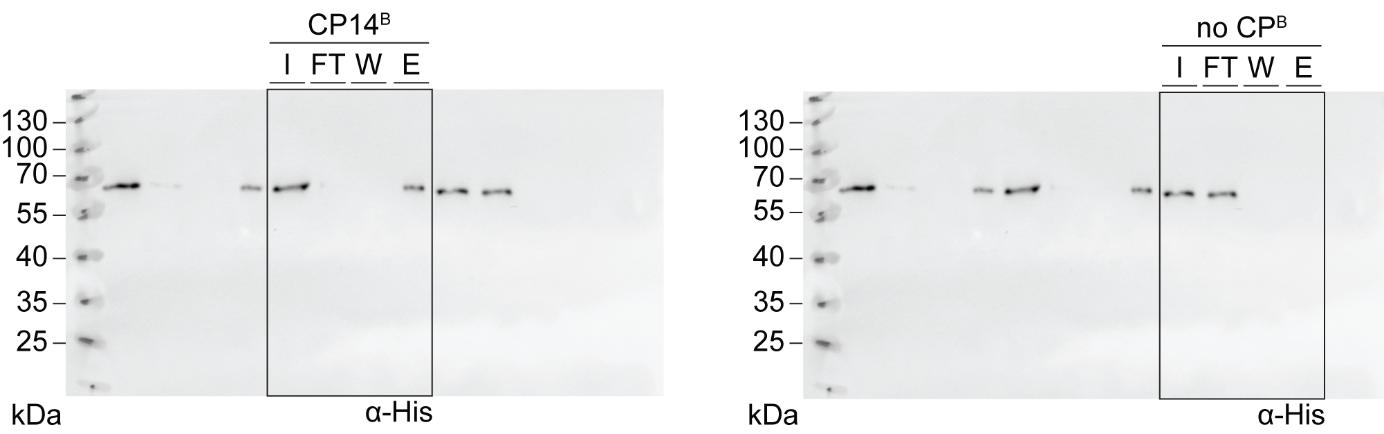


| Figure 2 | d |  |  |  |
| --- | --- | --- | --- | --- |


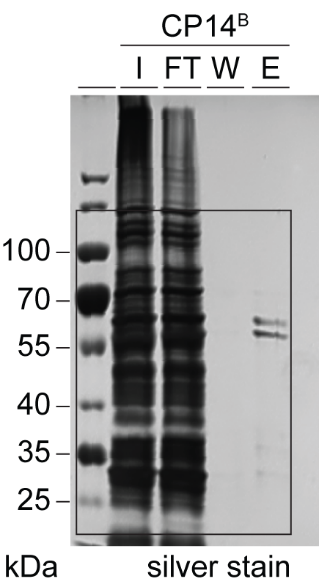

Supplement: Figure 2—source data 1. [file elife-67732-fig2-data1.docx]
