# Supplementary material for: De novo macrocyclic peptides dissect energy coupling of a heterodimeric ABC transporter by multimode allosteric inhibition
Source: eLife. 2021 Apr 30;10:e67732. doi: 10.7554/eLife.67732 (PMC8116058; doi:10.7554/eLife.67732)
Supplement: Figure 2—figure supplement 1—source data 1. [file elife-67732-fig2-figsupp1-data1.docx]

| Figure 2 | supplemental figure 1 |  | a |
| --- | --- | --- | --- |


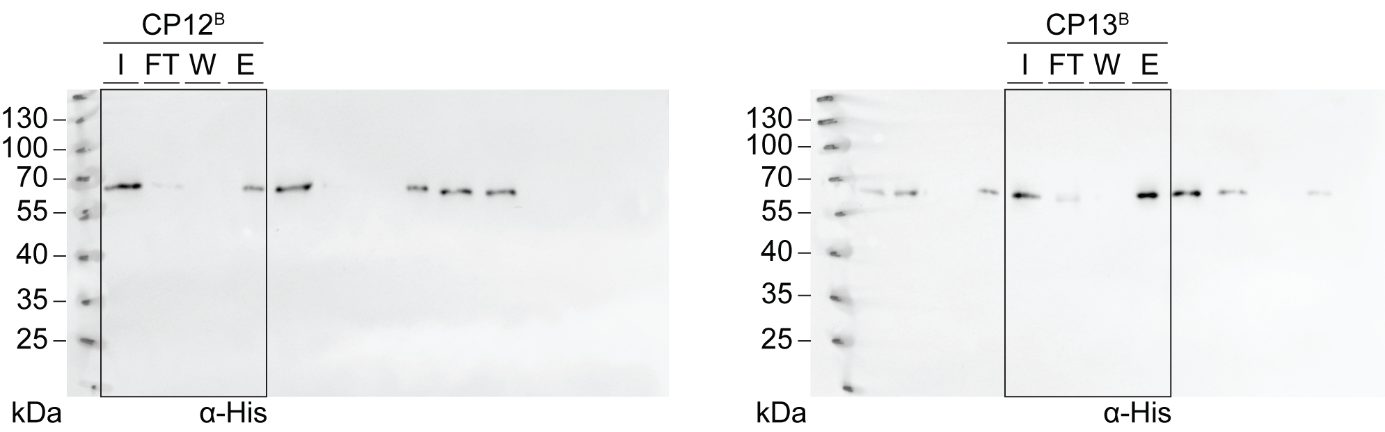


| Figure 2 | supplemental figure 1 |  | b |
| --- | --- | --- | --- |


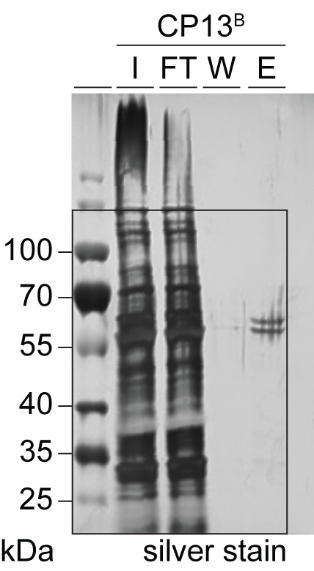


| Figure 2 | supplemental figure 1 | |  | d |
| --- | --- | --- | --- | --- |
|  |  |  |  |  |
|  |  |  | Fluorescence anisotropy | |
| CP6F |  |  | Mean | SD |
|  |  |  |  |  |
| CPF |  |  | 0.0222 | 0.0004 |
| TmrAB |  |  | 0.1093 | 0.0017 |
| SDS |  |  | 0.0146 | 0.0008 |
|  |  |  |  |  |
|  |  |  | Fluorescence anisotropy | |
| CP12F |  |  | Mean | SD |
|  |  |  |  |  |
| CPF |  |  | 0.0250 | 0.0006 |
| TmrAB |  |  | 0.1604 | 0.0011 |
| SDS |  |  | 0.0265 | 0.0006 |
|  |  |  |  |  |
|  |  |  | Fluorescence anisotropy | |
| CP13F |  |  | Mean | SD |
|  |  |  |  |  |
| CPF |  |  | 0.0252 | 0.0005 |
| TmrAB |  |  | 0.1217 | 0.0004 |
| SDS |  |  | 0.0310 | 0.0014 |
|  |  |  |  |  |
|  |  |  | Fluorescence anisotropy | |
| CP14F |  |  | Mean | SD |
|  |  |  |  |  |
| CPF |  |  | 0.0267 | 0.0008 |
| TmrAB |  |  | 0.1410 | 0.0065 |
| SDS |  |  | 0.0311 | 0.0011 |
