# Supplementary material for: De novo macrocyclic peptides dissect energy coupling of a heterodimeric ABC transporter by multimode allosteric inhibition
Source: eLife. 2021 Apr 30;10:e67732. doi: 10.7554/eLife.67732 (PMC8116058; doi:10.7554/eLife.67732)
Supplement: Figure 3—source data 1. [file elife-67732-fig3-data1.docx]

| Figure 3 | b |  |  |  |
| --- | --- | --- | --- | --- |
|  |  |  |  |  |
|  |  |  | ATP hydrolysis | |
|  |  |  | mmol Pi (g*min)^-1 | |
|  |  |  | Mean | SD |
|  |  |  |  |  |
| - |  |  | 1.49 | 0.23 |
| Autohydrolysis | |  | 0.62 | 0.02 |
| EDTA |  |  | 0.60 | 0.02 |
| CP6F |  |  | 0.54 | 0.07 |
| CP12F |  |  | 0.37 | 0.02 |
| CP13F |  |  | 0.39 | 0.04 |
| CP14F |  |  | 0.39 | 0.07 |

| Figure 3 | c |  |  |  |
| --- | --- | --- | --- | --- |
|  |  |  |  |  |
|  |  |  | Transported peptide | |
|  |  |  | µmol (g*min)^-1 | |
| ATP |  |  | Mean | SD |
|  |  |  |  |  |
| C4F |  |  | 1.64 | 0.17 |
| C4F/DMSO |  |  | 1.67 | 0.20 |
| CP6F |  |  | 0.33 | 0.01 |
| CP12F |  |  | 0.44 | 0.18 |
| CP13F |  |  | 0.33 | 0.04 |
| CP14F |  |  | 0.26 | 0.02 |
|  |  |  |  |  |
|  |  |  | Transported peptide | |
|  |  |  | µmol (g*min)^-1 | |
| ADP |  |  | Mean | SD |
|  |  |  |  |  |
| C4F |  |  | 0.00 | 0.00 |
| C4F/DMSO |  |  | 0.00 | 0.00 |
| CP6F |  |  | 0.38 | 0.01 |
| CP12F |  |  | 0.55 | 0.07 |
| CP13F |  |  | 0.28 | 0.01 |
| CP14F |  |  | 0.25 | 0.05 |

| Figure 3 | d |  |  |  |
| --- | --- | --- | --- | --- |
|  |  |  |  |  |
|  |  |  | Transported C4F peptide | |
|  |  |  | µmol (g*min)^-1 | |
| ATP |  |  | Mean | SD |
|  |  |  |  |  |
| - |  |  | 1.66 | 0.09 |
| CP6F |  |  | 0.29 | 0.03 |
| CP12F |  |  | 0.41 | 0.04 |
| CP13F |  |  | 0.41 | 0.06 |
| CP14F |  |  | 0.41 | 0.02 |
|  |  |  |  |  |
|  |  |  | Transported C4F peptide | |
|  |  |  | µmol (g*min)^-1 | |
| ADP |  |  | Mean | SD |
|  |  |  |  |  |
| - |  |  | 0.01 | 0.00 |
| CP6F |  |  | 0.37 | 0.05 |
| CP12F |  |  | 0.41 | 0.02 |
| CP13F |  |  | 0.25 | 0.04 |
| CP14F |  |  | 0.15 | 0.02 |
