# Supplementary material for: De novo macrocyclic peptides dissect energy coupling of a heterodimeric ABC transporter by multimode allosteric inhibition
Source: eLife. 2021 Apr 30;10:e67732. doi: 10.7554/eLife.67732 (PMC8116058; doi:10.7554/eLife.67732)
Supplement: Figure 4—source data 1. [file elife-67732-fig4-data1.docx]

| Figure 4 | a |  |  |  |  |
| --- | --- | --- | --- | --- | --- |
|  |  |  |  |  |  |
|  |  |  | Bound CP6F |  |  |
|  |  |  | Fluorescence anisotropy | |  |
|  |  |  | Mean | SD |  |
|  |  |  |  |  |  |
| - |  |  | 0.022 | 0.000 |  |
| TmrAB |  |  | 0.111 | 0.001 |  |
| C4ATTO655 |  |  | 0.109 | 0.002 |  |
| R9LQK |  |  | 0.101 | 0.001 |  |
|  |  |  |  |  |  |
|  |  |  | Bound CP12F | |  |
|  |  |  | Fluorescence anisotropy | |  |
|  |  |  | Mean | SD |  |
|  |  |  |  |  |  |
| - |  |  | 0.025 | 0.001 |  |
| TmrAB |  |  | 0.168 | 0.001 |  |
| C4ATTO655 |  |  | 0.160 | 0.001 |  |
| R9LQK |  |  | 0.155 | 0.001 |  |
|  |  |  |  |  |  |
|  |  |  | Bound CP13F | |  |
|  |  |  | Fluorescence anisotropy | |  |
|  |  |  | Mean | SD |  |
|  |  |  |  |  |  |
| - |  |  | 0.025 | 0.000 |  |
| TmrAB |  |  | 0.127 | 0.001 |  |
| C4ATTO655 |  |  | 0.122 | 0.000 |  |
| R9LQK |  |  | 0.123 | 0.001 |  |
|  |  |  |  |  |  |
|  |  |  | Bound CP14F | |  |
|  |  |  | Fluorescence anisotropy | |  |
|  |  |  | Mean | SD |  |
|  |  |  |  |  |  |
| - |  |  | 0.027 | 0.001 |  |
| TmrAB |  |  | 0.141 | 0.006 |  |
| C4ATTO655 |  |  | 0.143 | 0.000 |  |
| R9LQK |  |  | 0.140 | 0.002 |  |

| Figure 4 | b |  |  |  |  |
| --- | --- | --- | --- | --- | --- |
|  |  |  |  |  |  |
|  |  |  | Bound C4F peptide | |  |
|  |  |  | Fluorescence anisotropy | |  |
|  |  |  | Mean | SD |  |
|  |  |  |  |  |  |
| - |  |  | 0.023 | 0.000 |  |
| C4F |  |  | 0.129 | 0.001 |  |
| C4ATTO655 |  |  | 0.045 | 0.000 |  |
| CP6B |  |  | 0.124 | 0.001 |  |
| CP12B |  |  | 0.128 | 0.002 |  |
| CP13B |  |  | 0.141 | 0.000 |  |
| CP14B |  |  | 0.136 | 0.001 |  |

| Figure 4 | c |  |  |  |
| --- | --- | --- | --- | --- |
|  |  |  |  |  |
|  |  |  | Signal |  |
|  |  |  | cpm*10^3 |  |
|  |  |  | Mean | SD |
| ATP binding |  |  |  |  |
| - |  |  | 5.06 | 0.22 |
| CP6B |  |  | 4.22 | 0.13 |
| CP12B |  |  | 5.02 | 0.05 |
| CP13B |  |  | 6.33 | 0.27 |
| CP14B |  |  | 5.10 | 0.12 |
|  |  |  |  |  |
|  |  |  | Signal |  |
|  |  |  | cpm*10^3 |  |
|  |  |  | Mean | SD |
| Background |  |  |  |  |
| - |  |  | 0.00 | 0.04 |
| CP6B |  |  | -0.04 | 0.04 |
| CP12B |  |  | 0.08 | 0.03 |
| CP13B |  |  | 0.19 | 0.03 |
| CP14B |  |  | 0.08 | 0.03 |
