# Supplementary material for: De novo macrocyclic peptides dissect energy coupling of a heterodimeric ABC transporter by multimode allosteric inhibition
Source: eLife. 2021 Apr 30;10:e67732. doi: 10.7554/eLife.67732 (PMC8116058; doi:10.7554/eLife.67732)
Supplement: Figure 5—source data 1. [file elife-67732-fig5-data1.docx]

| Figure 5 | a |  |  |  |
| --- | --- | --- | --- | --- |
|  |  |  |  |  |
| IF |  |  | Bound CP6F |  |
| TmrAB |  |  | % |  |
| µM |  |  | Mean | SD |
|  |  |  |  |  |
| 0.000 |  |  | 0.000 | 0.001 |
| 0.005 |  |  | 1.520 | 0.692 |
| 0.050 |  |  | 28.686 | 1.566 |
| 0.250 |  |  | 66.724 | 0.674 |
| 0.500 |  |  | 80.637 | 0.681 |
| 1.000 |  |  | 88.296 | 0.819 |
|  |  |  |  |  |
| OF |  |  | Bound CP6F |  |
| TmrAB |  |  | % |  |
| µM |  |  | Mean | SD |
|  |  |  |  |  |
| 0.000 |  |  | 0.000 | 0.000 |
| 0.300 |  |  | 28.909 | 2.497 |
| 0.600 |  |  | 47.775 | 2.446 |
| 1.200 |  |  | 64.082 | 2.466 |
| 2.400 |  |  | 77.529 | 2.405 |
| 5.000 |  |  | 88.889 | 2.413 |
| 10.000 |  |  | 92.791 | 2.387 |

| Figure 5 | b |  |  |  |
| --- | --- | --- | --- | --- |
|  |  |  |  |  |
| IF |  |  | Bound CP13F | |
| TmrAB |  |  | % |  |
| µM |  |  | Mean | SD |
|  |  |  |  |  |
| 0.000 |  |  | 0.000 | 0.881 |
| 0.005 |  |  | 10.053 | 0.629 |
| 0.050 |  |  | 59.312 | 1.243 |
| 0.100 |  |  | 69.899 | 0.792 |
| 0.250 |  |  | 85.320 | 0.648 |
| 0.500 |  |  | 92.645 | 0.625 |
| 1.000 |  |  | 97.894 | 0.850 |
|  |  |  |  |  |
| OF |  |  | Bound CP13F | |
| TmrAB |  |  | % |  |
| µM |  |  | Mean | SD |
|  |  |  |  |  |
| 0.000 |  |  | 0.000 | 0.000 |
| 0.005 |  |  | 32.371 | 1.455 |
| 0.050 |  |  | 89.190 | 0.905 |
| 0.100 |  |  | 91.388 | 1.106 |
| 0.250 |  |  | 98.514 | 1.453 |
| 0.500 |  |  | 99.613 | 0.785 |
| 1.000 |  |  | 92.828 | 6.954 |

| Figure 5 | d |  |  |  |
| --- | --- | --- | --- | --- |
|  |  |  |  |  |
| ATP |  |  | Occluded nucleotides | |
|  |  |  | % |  |
|  |  |  | Mean | SD |
|  |  |  |  |  |
| 4 °C |  |  | 10.87 | 0.60 |
| Vanadate |  |  | 62.96 | 3.58 |
| CP6 |  |  | 6.76 | 0.40 |
| CP12 |  |  | 7.52 | 0.39 |
| CP13 |  |  | 55.84 | 2.81 |
| CP14 |  |  | 88.51 | 3.00 |
|  |  |  |  |  |
|  |  |  |  |  |
| ADP |  |  | Occluded nucleotides | |
|  |  |  | % |  |
|  |  |  | Mean | SD |
|  |  |  |  |  |
| 4 °C |  |  | 1.21 | 0.08 |
| Vanadate |  |  | 56.67 | 3.07 |
| CP6 |  |  | 2.66 | 0.46 |
| CP12 |  |  | 3.67 | 0.46 |
| CP13 |  |  | 10.47 | 0.44 |
| CP14 |  |  | 8.19 | 0.38 |
