# Supplementary material for: De novo macrocyclic peptides dissect energy coupling of a heterodimeric ABC transporter by multimode allosteric inhibition
Source: eLife. 2021 Apr 30;10:e67732. doi: 10.7554/eLife.67732 (PMC8116058; doi:10.7554/eLife.67732)
Supplement: Figure 5—figure supplement 1—source data 1. [file elife-67732-fig5-figsupp1-data1.docx]

| Figure 5 | supplemental figure 1 | | a |  |
| --- | --- | --- | --- | --- |
|  |  |  |  |  |
|  |  |  |  |  |
| IF |  |  | Bound CP12F | |
| TmrAB |  |  | % |  |
| µM |  |  | Mean | SD |
|  |  |  |  |  |
| 0.000 |  |  | 0.000 | 0.000 |
| 0.005 |  |  | 2.399 | 0.588 |
| 0.050 |  |  | 19.602 | 0.493 |
| 0.100 |  |  | 39.165 | 0.484 |
| 0.250 |  |  | 71.094 | 0.630 |
| 0.500 |  |  | 81.978 | 0.552 |
| 0.750 |  |  | 84.923 | 0.568 |
| 1.000 |  |  | 85.762 | 0.469 |
| 1.500 |  |  | 87.224 | 0.663 |
|  |  |  |  |  |
| OF |  |  | Bound CP12F | |
| TmrAB |  |  | % |  |
| µM |  |  | Mean | SD |
|  |  |  |  |  |
| 0.0 |  |  | 0.000 | 0.000 |
| 0.1 |  |  | 22.322 | 0.713 |
| 0.3 |  |  | 45.836 | 0.988 |
| 0.6 |  |  | 63.435 | 0.805 |
| 1.2 |  |  | 77.156 | 0.636 |
| 2.4 |  |  | 87.497 | 0.705 |
| 5.0 |  |  | 93.289 | 0.974 |
|  |  |  |  |  |
|  |  |  |  |  |
| IF |  |  | Bound CP14F | |
| TmrAB |  |  | % |  |
| µM |  |  | Mean | SD |
|  |  |  |  |  |
| 0.000000 |  |  | 0.000 | 0.104 |
| 0.000005 |  |  | 0.954 | 0.208 |
| 0.001000 |  |  | 0.978 | 0.169 |
| 0.005000 |  |  | 2.470 | 0.305 |
| 0.010000 |  |  | 7.410 | 0.127 |
| 0.050000 |  |  | 25.654 | 0.454 |
| 0.100000 |  |  | 60.455 | 0.344 |
| 0.200000 |  |  | 72.585 | 0.838 |
| 0.400000 |  |  | 76.033 | 0.314 |
|  |  |  |  |  |
| OF |  |  | Bound CP14F | |
| TmrAB |  |  | % |  |
| µM |  |  | Mean | SD |
|  |  |  |  |  |
| 0.001 |  |  | 1.491 | 0.290 |
| 0.005 |  |  | 4.069 | 0.270 |
| 0.050 |  |  | 41.344 | 0.277 |
| 0.100 |  |  | 74.905 | 0.918 |
| 0.200 |  |  | 85.570 | 0.540 |
| 0.400 |  |  | 87.819 | 0.325 |
| 0.800 |  |  | 88.678 | 0.300 |

| Figure 5 | supplemental figure 1 | | b |  |
| --- | --- | --- | --- | --- |
|  |  |  |  |  |
|  |  |  | Bound CPF |  |
|  |  |  | Fluorescence anisotropy | |
| CP6F |  |  | Mean | SD |
|  |  |  |  |  |
| 4 °C |  |  | 0.019 | 0.001 |
| TmrAB. 4 °C |  |  | 0.143 | 0.000 |
| TmrAB. 45 °C | |  | 0.132 | 0.001 |
| TmrAB. ADP. 45 °C | |  | 0.129 | 0.000 |
| TmrAB. ATP. 45 °C | |  | 0.050 | 0.001 |
|  |  |  |  |  |
|  |  |  | Bound CPF |  |
|  |  |  | Fluorescence anisotropy | |
| CP12F |  |  | Mean | SD |
|  |  |  |  |  |
| 4 °C |  |  | 0.023 | 0.000 |
| TmrAB. 4 °C |  |  | 0.127 | 0.001 |
| TmrAB. 45 °C | |  | 0.130 | 0.000 |
| TmrAB. ADP. 45 °C | |  | 0.122 | 0.000 |
| TmrAB. ATP. 45 °C | |  | 0.069 | 0.001 |
|  |  |  |  |  |
|  |  |  | Bound CPF |  |
|  |  |  | Fluorescence anisotropy | |
| CP13F |  |  | Mean | SD |
|  |  |  |  |  |
| 4 °C |  |  | 0.023 | 0.000 |
| TmrAB. 4 °C |  |  | 0.055 | 0.000 |
| TmrAB. 45 °C | |  | 0.051 | 0.001 |
| TmrAB. ADP. 45 °C | |  | 0.050 | 0.001 |
| TmrAB. ATP. 45 °C | |  | 0.048 | 0.000 |
|  |  |  |  |  |
|  |  |  | Bound CPF |  |
|  |  |  | Fluorescence anisotropy | |
| CP14F |  |  | Mean | SD |
|  |  |  |  |  |
| 4 °C |  |  | 0.023 | 0.000 |
| TmrAB. 4 °C |  |  | 0.134 | 0.001 |
| TmrAB. 45 °C | |  | 0.132 | 0.001 |
| TmrAB. ADP. 45 °C | |  | 0.131 | 0.001 |
| TmrAB. ATP. 45 °C | |  | 0.127 | 0.001 |
