# Supplementary material for: De novo macrocyclic peptides dissect energy coupling of a heterodimeric ABC transporter by multimode allosteric inhibition
Source: eLife. 2021 Apr 30;10:e67732. doi: 10.7554/eLife.67732 (PMC8116058; doi:10.7554/eLife.67732)
Supplement: Figure 6—source data 1. [file elife-67732-fig6-data1.docx]

| Figure 6 | b |  |  |  |
| --- | --- | --- | --- | --- |
|  |  |  |  |  |
|  |  |  | Peptides/liposome | |
|  |  |  | Mean | SD |
|  |  |  |  |  |
| ATP |  |  | 84.57 | 0.93 |
| ADP |  |  | 3.52 | 0.43 |
| CP6F |  |  | 4.74 | 0.61 |
| CP12F |  |  | 4.93 | 0.25 |
| CP13F |  |  | 13.69 | 0.21 |
| CP14F |  |  | 38.81 | 0.24 |

| Figure 6 | c |  |  |  |
| --- | --- | --- | --- | --- |
|  |  |  |  |  |
| Without CP |  |  |  |  |
| Time |  |  | Peptides/liposome | |
| min |  |  | Mean | SD |
|  |  |  |  |  |
| 0 |  |  | 0.00 |  |
| 1 |  |  | 33.27 | 0.53 |
| 2 |  |  | 84.85 | 0.84 |
|  |  |  |  |  |
| CP13F |  |  |  |  |
| Time |  |  | Peptides/liposome | |
| min |  |  | Mean | SD |
|  |  |  |  |  |
| 1 |  |  | -0.42 | 0.28 |
| 3 |  |  | 2.53 | 0.32 |
| 5 |  |  | 3.33 | 0.33 |
| 10 |  |  | 5.08 | 0.36 |
| 20 |  |  | 4.95 | 0.30 |
| 40 |  |  | 5.22 | 0.32 |
|  |  |  |  |  |
| CP14F |  |  |  |  |
| Time |  |  | Peptides/liposome | |
| min |  |  | Mean | SD |
|  |  |  |  |  |
| 1 |  |  | 0.03 | 0.33 |
| 3 |  |  | 6.21 | 0.26 |
| 5 |  |  | 14.91 | 0.44 |
| 10 |  |  | 24.30 | 0.62 |
| 20 |  |  | 38.29 | 0.71 |
| 40 |  |  | 48.57 | 0.67 |

| Figure 6 | d |  |  |  |  |
| --- | --- | --- | --- | --- | --- |
|  |  |  |  |  |  |
| Without CP |  |  |  |  |  |
| Time |  |  | Mean fluorescence intensity * 10^3 | | |
| min |  |  | Mean | SD |  |
|  |  |  |  |  |  |
| 1 |  |  | 2.29 | 0.04 |  |
| 2 |  |  | 5.83 | 0.06 |  |
| 3 |  |  | 10.49 | 0.07 |  |
| 5 |  |  | 18.11 | 0.16 |  |
| 10 |  |  | 30.27 | 0.13 |  |
| 20 |  |  | 40.72 | 0.11 |  |
| 40 |  |  | 51.44 | 0.25 |  |
|  |  |  |  |  |  |
| CP13F |  |  |  |  |  |
| Time |  |  | Mean fluorescence intensity * 10^3 | | |
| min |  |  | Mean | SD |  |
|  |  |  |  |  |  |
| 1 |  |  | -0.03 | 0.02 |  |
| 3 |  |  | 0.17 | 0.02 |  |
| 5 |  |  | 0.23 | 0.02 |  |
| 10 |  |  | 0.35 | 0.03 |  |
| 20 |  |  | 0.34 | 0.02 |  |
| 40 |  |  | 0.36 | 0.02 |  |
|  |  |  |  |  |  |
| CP14F |  |  |  |  |  |
| Time |  |  | Mean fluorescence intensity * 10^3 | | |
| min |  |  | Mean | SD |  |
|  |  |  |  |  |  |
| 1 |  |  | 0.00 | 0.02 |  |
| 3 |  |  | 0.43 | 0.02 |  |
| 5 |  |  | 1.02 | 0.03 |  |
| 10 |  |  | 1.67 | 0.04 |  |
| 20 |  |  | 2.63 | 0.05 |  |
| 40 |  |  | 3.34 | 0.05 |  |

| Figure 6 | e |  |  |  |
| --- | --- | --- | --- | --- |
|  |  |  |  |  |
| CP6F |  |  |  |  |
| Time |  |  | Peptides/liposome | |
| min |  |  | Mean | SD |
|  |  |  |  |  |
| 0 |  |  | 0.00 | 0.00 |
| 5 |  |  | 0.17 | 0.30 |
| 65 |  |  | 0.01 | 0.15 |
| 70 |  |  | 1.93 | 0.14 |
| 130 |  |  | 1.54 | 0.16 |
|  |  |  |  |  |
| CP12F |  |  |  |  |
| Time |  |  | Peptides/liposome | |
| min |  |  | Mean | SD |
|  |  |  |  |  |
| 0 |  |  | 0.00 | 0.00 |
| 5 |  |  | 1.11 | 0.23 |
| 65 |  |  | 1.17 | 0.30 |
| 70 |  |  | 1.70 | 0.22 |
| 130 |  |  | 1.01 | 0.23 |
|  |  |  |  |  |
| CP13F |  |  |  |  |
| Time |  |  | Peptides/liposome | |
| min |  |  | Mean | SD |
|  |  |  |  |  |
| 0 |  |  | 0.00 | 0.00 |
| 5 |  |  | 9.32 | 0.28 |
| 65 |  |  | 10.24 | 0.32 |
| 70 |  |  | 10.66 | 0.26 |
| 130 |  |  | 11.66 | 0.28 |
|  |  |  |  |  |
| CP14F |  |  |  |  |
| Time |  |  | Peptides/liposome | |
| min |  |  | Mean | SD |
|  |  |  |  |  |
| 0 |  |  | 0.00 | 0.00 |
| 5 |  |  | 36.27 | 0.52 |
| 65 |  |  | 37.68 | 0.52 |
| 70 |  |  | 44.92 | 0.70 |
| 130 |  |  | 48.07 | 0.66 |
