# Supplementary material for: De novo macrocyclic peptides dissect energy coupling of a heterodimeric ABC transporter by multimode allosteric inhibition
Source: eLife. 2021 Apr 30;10:e67732. doi: 10.7554/eLife.67732 (PMC8116058; doi:10.7554/eLife.67732)
Supplement: Figure 6—figure supplement 1—source data 1. [file elife-67732-fig6-figsupp1-data1.docx]

| Figure 6 | supplemental figure 1 | | c |  |  |
| --- | --- | --- | --- | --- | --- |
|  |  |  |  |  |  |
|  |  |  | Mean fluorescence intensity | | |
| Peptides/liposome | |  | Mean | SD |  |
|  |  |  |  |  |  |
| 1.24 |  |  | 104.37 | 38.60 |  |
| 4.96 |  |  | 358.70 | 41.02 |  |
| 10.78 |  |  | 701.70 | 38.48 |  |
| 42.16 |  |  | 3204.37 | 38.72 |  |
| 88.50 |  |  | 5937.37 | 43.81 |  |
